# Practical Notes and Formulæ

**Published:** 1878-07-20

**Authors:** 


Page(s) missing

WINE OF GENTIAN.

R.—Fluid ext. of gentian.........oz. ½.

Fluid ext. cinchonia..........oz. i.

Fluid ext. orange peel...........dr. ii.

Fluid ext. canella............dr. i.

Proof spirit................oz. 4½.

Sherry wine...........oz. xxxvi.

A pleasant stomachic bitter. Dose, one or two tablespoonsful.

ANTI-COLIC MIXTURE.

R.—Tinct. rhubarb..............oz. ii.
Tinct. capsicum—
Aromat. spts. ammonia, aa...dr. ii.
Deod. tinct. opium..........dr. i.
Oil peppermint............gtts. vi.
Water, q.s. for..............oz. vi.

Dose, one to two tablespoonsful, for an adult, every two to three hours. Adapted to colic pains and flatulent conditions from imperfect and feeble digestion.

SANTONINE POISONING.

Chloral hydrate is said to be an antidote to santonine, and ether inhalation is likewise recommended.

ASTHMA.

R.—Iodide potassium, } aa.... oz. ½.

Bromide potassium, }

Simple elixir..............ozs. x.

M. Dose, one teaspoonful every two hours during the paroxysm. May be given three times daily for a long period to ward off attacks, and is sometimes effectual in removing the disease entirely.

WHOOPING COUGH.

A writer (in *Med. Times*) says that the following is a specific in whooping cough:

R.—Picrate of ammonia..........gr. i.
Muriate of ammonia... .gr. xxiv.
Powdered ext. of liquorice....dr. i.
Water...................oz. iii.

M. Dose, a teaspoonful every three hours, and more or less according to age.

TINCTURE STOPPERS.

The unpleasant cementing of stoppers can be entirely prevented by rubbing the stoppers with a piece of paraffin, and giving them a turn in the neck of the bottle, so as to distribute a thin coating of paraffin all over. Renew two or three times a year.—*Phil. Drug. and Chem.*

DIAGNOSIS OF PREGNANCY.

The following general rule to the dignosis of pregnancy laid down by Prof. Goodell has the advantage of being couched in familiar terms: "When the neck of the uterus appears to you as hard as the end of your nose, pregnancy should not exist; if it appears to you as soft as your lips, the uterus probably contains a fœtus.—*Mich. Med. News.*

SPIRIT OF CHLOROFORM.

R.—Chloroform...........(troy) oz. j.

Alcohol (strong) .........oz. xii.

M. This is an excellent stimulating anodyne; and useful also as a liniment. Dose, one to three drachms.

CAMPHENYL.

Camphenyl is produced from coal tar. Is recommended as a substitute for carbolic acid. It claims to possess all the virtues and none of the dangerous qualities of carbolic acid.

DYSMENORRHŒA.

Dr. A. P. Brown, of Jefferson, Texas, recommends the following in dysmenorrhœa:

R.—Hyd. c. corrosive............gr. i.
Ferri et pot. tart..........gr. xx.
Tinct. stillingæ..............oz. iij.

M. Dose, half to a teaspoonful three times per day, accompanied with proper hygienic rules.

FOR CONSTIPATION.

R.—Fluid ext. cascara..........oz. ss.
Aq. cinnamon..............oz. jss.
Sac. alb...................dr. ij.

M. Dose, teaspoonful three times per day.—*Writer in Clinic.*

